# An analysis of COVID-19 clusters in India: Two case studies on Nizamuddin and Dharavi

**DOI:** 10.1186/s12889-021-10491-8

**Published:** 2021-03-31

**Authors:** Pooja Sengupta, Bhaswati Ganguli, Sugata SenRoy, Aditya Chatterjee

**Affiliations:** 1 International Management Institute, Kolkata, India; 2 Department of Statistics, University of Calcutta, Kolkata, India

**Keywords:** SARS-COV2, Close contact epidemiology, Cluster analysis

## Abstract

**Background:**

In this study we cluster the districts of India in terms of the spread of COVID-19 and related variables such as population density and the number of specialty hospitals. Simulation using a compartment model is used to provide insight into differences in response to public health interventions. Two case studies of interest from Nizamuddin and Dharavi provide contrasting pictures of the success in curbing spread.

**Methods:**

A cluster analysis of the worst affected districts in India provides insight about the similarities between them. The effects of public health interventions in flattening the curve in their respective states is studied using the individual contact SEIQHRF model, a stochastic individual compartment model which simulates disease prevalence in the susceptible, infected, recovered and fatal compartments.

**Results:**

The clustering of hotspot districts provide homogeneous groups that can be discriminated in terms of number of cases and related covariates. The cluster analysis reveal that the distribution of number of COVID-19 hospitals in the districts does not correlate with the distribution of confirmed COVID-19 cases. From the SEIQHRF model for Nizamuddin we observe in the second phase the number of infected individuals had seen a multitudinous increase in the states where Nizamuddin attendees returned, increasing the risk of the disease spread. However, the simulations reveal that implementing administrative interventions, flatten the curve. In Dharavi, through tracing, tracking, testing and treating, massive breakout of COVID-19 was brought under control.

**Conclusions:**

The cluster analysis performed on the districts reveal homogeneous groups of districts that can be ranked based on the burden placed on the healthcare system in terms of number of confirmed cases, population density and number of hospitals dedicated to COVID-19 treatment. The study rounds up with two important case studies on Nizamuddin basti and Dharavi to illustrate the growth curve of COVID-19 in two very densely populated regions in India. In the case of Nizamuddin, the study showed that there was a manifold increase in the risk of infection. In contrast it is seen that there was a rapid decline in the number of cases in Dharavi within a span of about one month.

## Background

The Corona Virus Disease 2019 (COVID-19) caused by the novel corona virus SARSCoV-2, started at Wuhan in the Hubei province of China and has spread with great speed around the world. More than a year has passed since and the virus is still wreaking havoc in many nations including especially the US and India. The objectives of the analysis presented in this paper are as follows:

- In this study we cluster the districts of India in terms of the spread of COVID-19 and related variables such as population density and the number of specialty hospitals.
- Simulation using a compartment model is used to provide insight into differences in response to public health interventions.
- Two case studies of interest from Nizamuddin and Dharavi provide contrasting pictures of the success in curbing spread of COVID-19.

As COVID-19 pandemic aggressively spreads across continents, vigorous public health responses are now being put in place in all the countries hit by the virus. Articles are being added to the expanding body of work on COVID-19 in *flattening* *the* *curve*. Many recent research papers have discussed the assessment of these containment policies through prediction of the path of COVID-19 cases in India, for different scenarios [1–5]. Some recent papers have discussed in detail the outbreak of the disease across the globe. The main underlying theme of most of these studies is to track and predict the path of the virus spread in the nation. Researchers have toiled to predict the time it will take the outbreak to subside. As a result different groups of researchers have made use of various modelling techniques for prediction and have come up with many interesting findings. For example, [6] have revealed that a reduction in the contact rate between uninfected and infected individuals by quarantining the susceptible individuals, can in effect reduce the basic reproduction number. Now, with the advent of the COVID-19 outbreak, governments all over the world have started enforcing interventions like increased quarantining, self-isolation, increased hospital facilities etc. In this paper we have made an endeavour to follow the path of the disease and predict the prevalent number of susceptible, infected, recovered, and fatal cases for the Indian population cohort. This is done through some simulations with the timely government interventions being put in place. Essentially the simulation helps us predict prevalence of the infections, numbers recovered and fatal cases through simulations using the SEIQHRF epidemic model, by moderating the parameters of the model according to the government interventions and emergency policies.

In this study we have performed a cluster analysis on the worst hit districts of India. With variables like number of confirmed COVID-19 cases, number of COVID-19 hospitals, and population density of the respective districts we have tried to classify different districts in India according to their homogeneity in nature due to these factors. The main objective of clustering in this study is to improve monitoring of the affected areas which will be useful in understanding seriousness of the spread of novel coronavirus (COVID-19) to revamp government policies, decisions, medical facilities (ventilators, testing kits, masks etc.), treatment etc. This in turn will help to reduce number of infected and deceased individuals. A similar clustering approach is used in a study carried out by [7].

In addition to clustering, a scenario analysis of the respective areas facilitate following the growth curve in the respective states. For that purpose, individuals have been divided into various groups, such as, susceptible, exposed, infected, infectious (but self isolated), hospitalized, recovered and dead (death but not hospitalized, from COVID-19). Various parameters used in the probabilistic model are, the number of per day exposure events (*act*) between infectious and susceptible individuals; chance of iinfection being spread at each exposure event between the infectious and susceptible; rate at which the symptomatic self isolate themselves; the per day rate of symptomatic people requiring hospitalisation; per day rate at which people requiring hospitalisation recover; and per day fatality rate for people needing hospitalisation but could not because the full capacity of the hospitals was occupied. Each of these groups of individuals are divided into these various compartments, and the transition rates of individuals into and out of these compartments are also taken into consideration in the model. The purpose of this method used is to look at different intervention experiments and follow the prevalence of COVID-19 (in terms of number of persons) with each passing day since the beginning of the epidemic. The research has been carried out for some of the Indian states where we find the maximum number of districts with high number of confirmed COVID-19 cases. The *act* parameter in this study, is adjusted for each state based on its population density. Thus, we follow the prevalence curve of the cohorts from the chosen states and evaluate the effectiveness of the interventions imposed by the government to curb the COVID-19 growth.

The rest of the paper is organized as follows: “Methods” section describes the SEIQHRF epidemic model and explains the parameters involved and explains the two clustering techniques used to classify districts. “Results” section reports the district-wise cluster analysis results. “Discussion” section discusses the case of Nizamuddin basti in Delhi,where a religious gathering exposed thousands to this virus and caused a second wave of infection in states when Nizamuddin attendees returned home. Here we discuss the results from fitting the SEIQHRF model. This section also discusses the case of Dharavi, one of Asia’s largest slums with very high population density. “Conclusion” section enumerates the findings of this study and concluding remarks.

## Methods

### Clustering

Two of the most commonly used algorithms in clustering are the hierarchical and the k-means algorithm. In this study we have used both these techniques of clustering technique due to their easily interpretable visualization and intuitive interpretation. One of the most relevant characteristics of hierarchical clustering is the fact that the user does not need to provide the number of clusters beforehand. Based on the variable upon which clustering is done, the observations are clustered into optimum number of groups. However a disadvantage of the method is that it has a high computational complexity when the number of observations to classify is very high. Thus using it on all 640 districts in India is quite cumbersome. So we have chosen the top 50 worst affected districts with maximum number of confirmed COVID-19 cases. The methodology used to build the hierarchical clustering, as also used in [8], is the following:

1. Pairwise distance between all the hotspot districts is calculated using *D*_*i*_(*S*_*A*_,*S*_*B*_), for a particular value of the parameter *α*. This distance matrix is symmetrical and has a null diagonal, which will be important to analyze the similarity between the different districts.
2. Searching through the distance matrix, we select the two most similar two districts.
3. These two districts are joined then to produce a new group that now has at least two districts.
4. The distance matrix is then updated by calculating the distances between the new cluster and all other clusters.
5. Step 2 is repeated until all districts belong to a group.

The k-means algorithm is one of the most popular clustering technique that divides the data samples into pre-defined distinct subgroups, where each data point belongs to just one group. It keeps data points within a cluster similar to each other and also tries to maximize the heterogeneity between two clusters. A cluster refers to a set of information aggregated together owing to certain similarities. Unlike hierarchical clustering in k-means the number of cluster is prefixed. The optimum number of clusters, say *k*, is obtained by using an elbow plot. From the dataset the method identifies *k* centroids, and then assigns every data point to the cluster it is closest to, keeping the centroids as small as possible. The clustering stops when either:

1. The centroids have more or less become constant and there is no change in their values.
2. The number of iterations defined has been reached.

In our study, data on confirmed COVID-19 cases, population density and number of COVID-19 hospitals in each districts is used to get the set of centroids. Although before the *k*-means clustering is performed the variables were standardized so that they are all on the same scale.

### SEIQHRF model

**Fig. 1:**
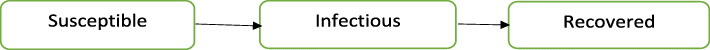
SIR model flowchart- This figure represents the different compartments of the model and the arrows represent the direction of flow of individuals from one to the other. The SIR model predicts the prevalence of susceptible, infected and recovered individuals

One of the popular models used for predicting the course of an epidemic such as COVID-19, is the Susceptible-Infectious-Recovered (SIR) model ([9–11]), [12]. In this model, the population is segregated into three separate compartments, i.e. Susceptible (S), Infectious (I) and Removal (R), where transition rates between the compartments is defined beforehand. Figure 1 is a representation of the three compartments in SIR model. In order to describe the per day rate at which individuals move in or out of the compartments, two equations are used. It is by solving these equations that we get the prevalence in each compartment.

**Fig. 2:**
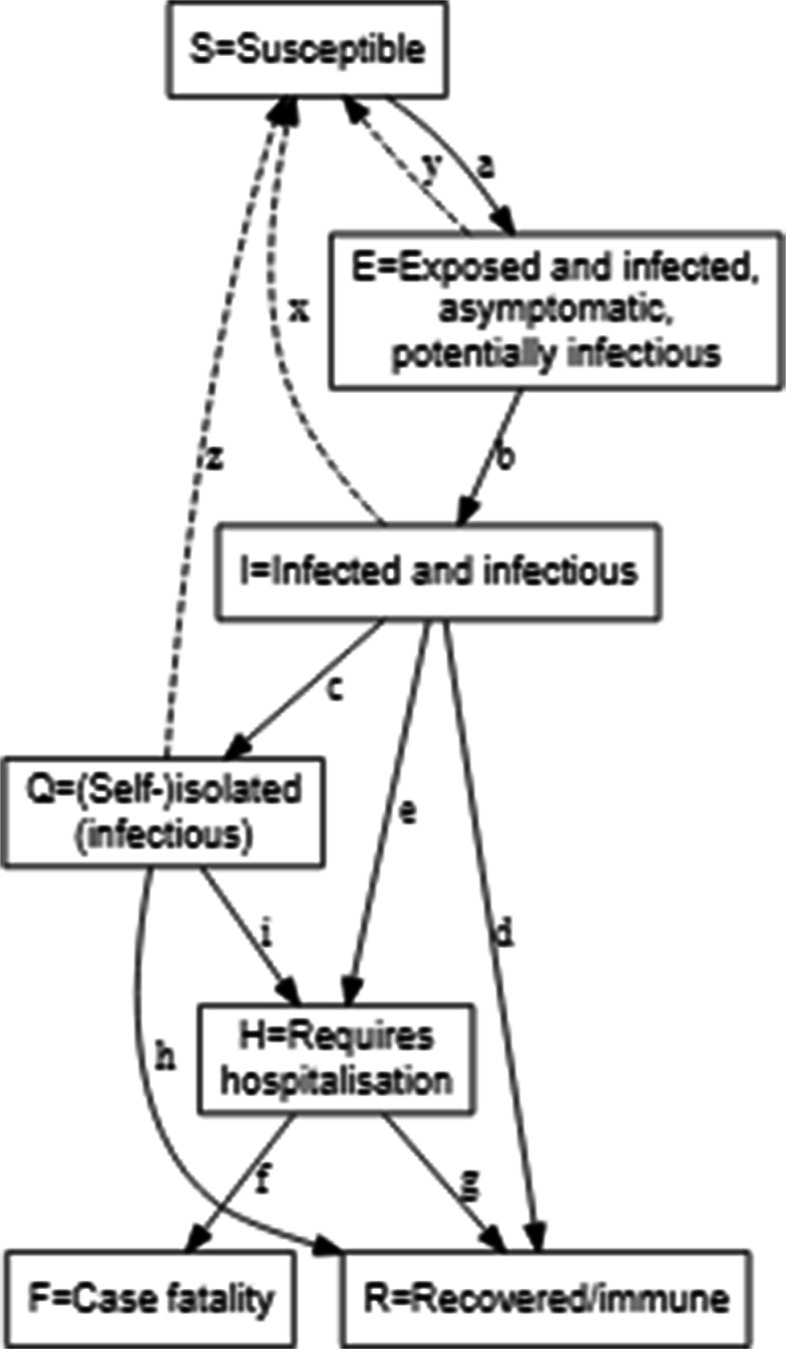
SEIQHRF model flowchart-This figure represents the various compartments and the arrows represent the direction of movement of individuals from one to the other

The SEIR model, a variant of the SIR model, is one which includes a new compartment called Exposed (E), [13–15]. However, more recently an extension of the SEIR model has been proposed by [4, 16], where apart from the three compartments, Susceptible (S), Infectious (I), Exposed (E) and Removal (R), two other compartments are included. These compartments are Quarantine (Q) and Hospitalization (H). The compartment H is supposed to take into account the healthcare capability of a state/district. In this new model the Removal (R) stage is divided into Recovery (denoted by R) compartment and fatality (denoted by F) compartment. The dynamic nature of the model is due to the fact that the number of individuals in each compartment may change over time. The flowchart showing the various compartments in the model and the arrows depict the direction of movement of individuals into and out of them is shown in Fig. 2 [16]. The following Table 1, provides a description of individual compartments.

**Table 1:** Description of compartments in SEIQHRF model

State	Indicator	Functional Definition
Susceptible	S	Susceptible to COVID-19
Exposed	E	Exposed and infected, but not yet symptomatic, have the potential to become infectious
Infected	I	Infected symptomatic as well as infectious
Quarantined	Q	Infectious and self-isolated i.e. individuals who are isolated and hence do not come in contact with the susceptible popualtion)
Hospitalized	H	Those that require hospitalization and would normally be hospitalized if capacity was available
Recovered	R	Recovered, assumed to be immune from further infection. However, repeat infection is possible, but chances are low and for the time being assumed disregardable.
Fatality	F	Case fatality (death exclusively due to COVID-19 and of no other cause.

With the introduction of more compartments into the SIR and SEIR models, the systems of equations that should be solved to get the prevalence, become very complicated. Thus, in case of SEIQHRF, probabilistic compartment models are developed. Probabilistic models help simulate individuals in a population, which allows them to move between compartments and make it easy to specify various probabilities of passing on infection between the individuals in the different compartments, such as moving from infectious to susceptible. The probabilistic model can be further improved to adequately model real-life scenarios. We have thus used the model to explore the effects numerous public health interventions and policies through scenario analyses, [5].

The extensions of SEIR model adds a number of compartments in the analysis. The *E* compartment is for the individuals exposed to the virus and infected with it, but are still asymptomatic with the potential of becoming infectious. This is also true for the traditional SEIR models, but the normal assumption is that the asymptomatic infected do not spread the infection to others. But that condition was relaxed in [16] due to increasing evidence in support that most infections are being spread during the incubation days when the carrier is asymptomatic. This evidence also supports the flowchart in Fig. 2, where there is a movement of individuals from the *I* infected symptomatic compartment to *Q*, quarantine compartment. But the individuals in the *E* compartment only move to the *Q* compartment when they have gone through compartment *I* i.e. only when they have shown some symptoms of the infection.

Similarly, the *H* compartment represents those that need hospitalisation. There’s a parameter *hosp*.*cap* that specifies the hospital capacity, and it is possible to indicate increased mortality rates for those who need hospitalisation in the *H* compartment but cannot get such care because the total *hosp*.*cap* has been exhausted. Related to this, the *F* compartment shows the case fatalities; i.e. the deaths in COVID-19 cases due to the virus. There are also some parameters for background death rates due to other causes among the susceptible (defined as the crude death rate for India). Also note that case fatalities are restricted only to occur among those in the H compartment, that is, those requiring hospitalisation (irrespective of whether they can get it or not). A similar method for modelling spread of COVID-19 has been used by [5].

In the present study we make an endeavour to fit the SEIQHRF model mentioned in Fig. 2 to COVID-19 outbreak data for some states in India where COVID-19 has been rampant. The purpose of this model is two-fold. First, the resulting simulations are used to study in detail the impact of lockdown on the epidemic. Second, we perform a what -if analysis for strategies to impose and relax lockdown at the most opportune time.

### Choice of parameters

The timeline used for this study is the beginning of the second phase of lockdown in India, primarily because the spread of cases from the Nizamuddin cluster became prominent towards the beginning of April and the first case in Dharavi slum was also reported on the 1st of April. Thus using 15th April, the start of lockdown phase 2 in India as the starting point of this study. We obtain the best fit to the observed cases and fatality data of COVID-19 by using the following set of parameters, which are represented in Table 2.

**Table 2:** Choice of parameters in SEIQHRF model

Serial No.	Parameter	Values
1	Number of per day exposure events between the symptomatic infected and susceptible	**20** (as in both Nizamuddin and Dharavi, the population density was quite high, making social distancing impossible.
2	Probability that an infection is passed from infectious to susceptible	**0.03**
3	Number of per day exposure events between infectious and susceptible	**40** (as the testing rate was low number of exposures are quite high)
4	Probability of infection being passed from asymptomatic to susceptible	**0.01**
5	Number of exposures per day between the quarantined and susceptible	**2**
6	Probability of the infection passing from quarantined to susceptible	**0.02**
7	Rate at which symptomatic, infected enter quarantine per day	**1/5** (as both quarantining and advocating self quarantine started early in india, this rate is somewhat low)
8	Transition from infected or quarantined to hospitalized was done with a random sample with a sample fraction	**1/50** (assuming 20% of the symptomatic cases progress to severe or critical disease with an average illness duration of 15 days).
9	Available hospital beds for the susceptible who are infected	**40**
10	Daily death rate of susceptible	**7.3/1000/365** (based on CDR for India)
11	Daily death rate of asymptomatic	**20/1000/365** (as they are probably in early stages of infection)
12	Daily death rate of quarantined	**30/1000/365**
13	Daily death rate of infected	**30/100/365** (As deaths will occur in the H compartment which denotes the ones needing hospitalization, not necessarily hospitalised as all individuals may not seek care or have access)
14	Daily death rate among hospitalised	**80/365/1000.**

The values in bold represent the parameter values used for model fitting

The starting day for the simulation was taken as 15th April 2020 (when second phase of lockdown began). The initial susceptible population in Nizamuddin Basti was taken as 12,000 (it has a floating population of 2000-5000 per day and a population density approx 70,000 *km*^2^.) (Source: Aga Khan Developmental Network). Dharavi has a population of about 1,000,000. With a population density of over 277,136 per *km*^2^. As the incubation period of COVID-19 is about 5 days, the number of infected cases (I compartment) on 15th April was assumed to be the number of new cases 5 days thereafter, i.e. on 20th April. It is especially interesting that the E compartment of asymptomatic infectious individuals, who are accountable for increased case numbers even after improvement in healthcare facilities for symptomatic cases.

## Results

### Analysis of hotspot districts

With the recent updates on COVID-19 cases in India, it has become evident that some parts of the country are more affected than others. For instance, Maharashtra, Tamil Nadu, Delhi, Gujarat, Telangana, Karnataka and Uttar Pradesh are on the top of the list of most affected states in India. At a more disaggregate level, few districts of these states are more badly affected. The common trend in COVID-19 infection is that mostly the urban regions have been more affected than their rural counterparts. This is primarily due to the fact that the virus was introduced by travellers from abroad. The variables used in this study are described in detail in Table 3 below. The summary measures of population density and number of COVID-19 hospitals reveal a large variation between the districts, the former ranging from 161 to 27730 persons per sq.km. whereas the latter ranges from no hospitals to 62 hospitals in a district.(https://covidindia.org, https://www.census2011.co.in/district.php). These variations necessitate performing a cluster analysis to optimally classify the districts into homogeneous groups. Both hierarchical clustering and k-means clustering methods are done using the confirmed cases, population density and number of COVID-19 hospitals.

**Table 3:** Summary of descriptive measures of the variables

Variables	Description	Min	Mean	Median	Max	St. Dev
Confirmed	No.of confirmed cases	122	5651	1332	74252	13545.7
COVID hospitals	No. of COVID-19 dedicated hospitals	0	7.14	4	62	11.08
Density	Population density	161	3746.4	838.5	27730	7320.3

**Fig. 3:**
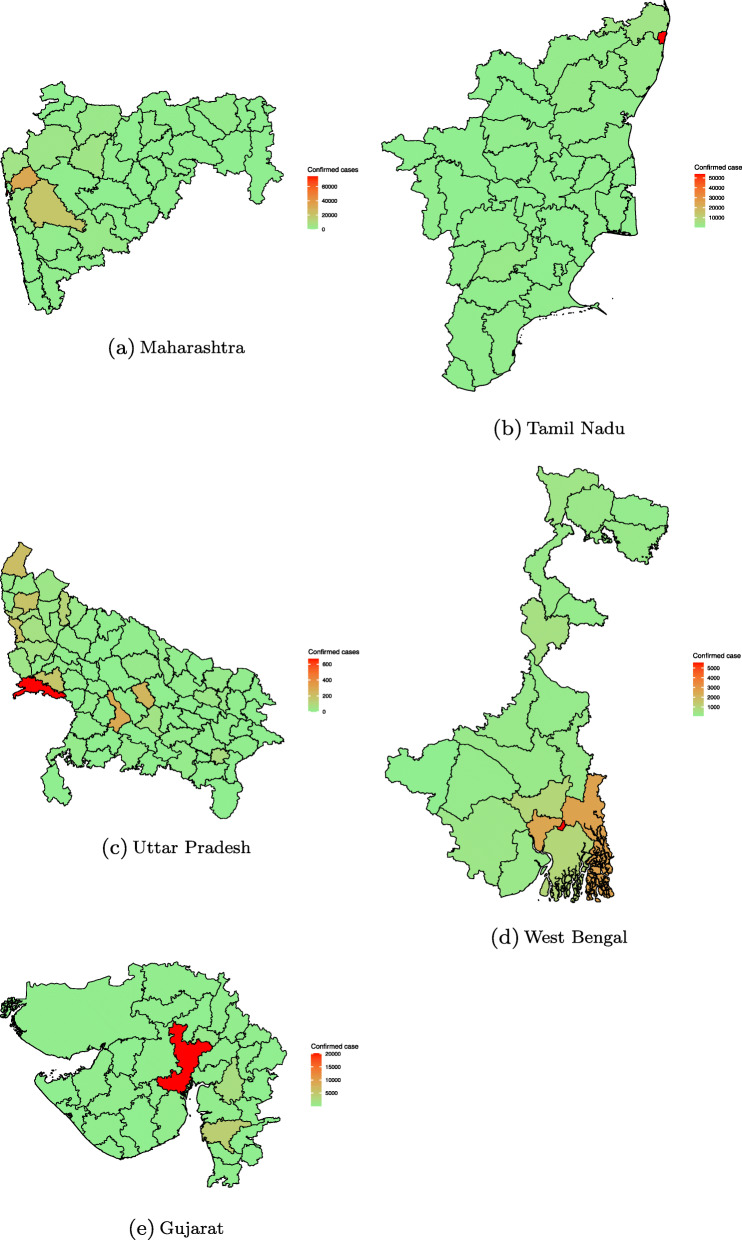
Distribution of COVID-19 cases in districts of selected states. These maps were created by the authors using R, packages *ggmap*, *maps*, *maptools* and *rgeos*

If we focus on the distribution of COVID-19 cases across states, the metropolitan areas are most affected in most of them. For Maharashtra (Fig. 3), the district marked in red is in the extreme west coastal region, Mumbai city and Mumbai Suburban. Similarly, in Gujarat, the most affected district is Ahmedabad. In Uttar Pradesh and Tamil Nadu the main hotspot districts are Agra and Chennai respectively. In West Bengal the main focus is on three districts, i.e. Kolkata, Howrah and North 24 Parganas (as shown by the red zones in the state map).

**Fig. 4:**
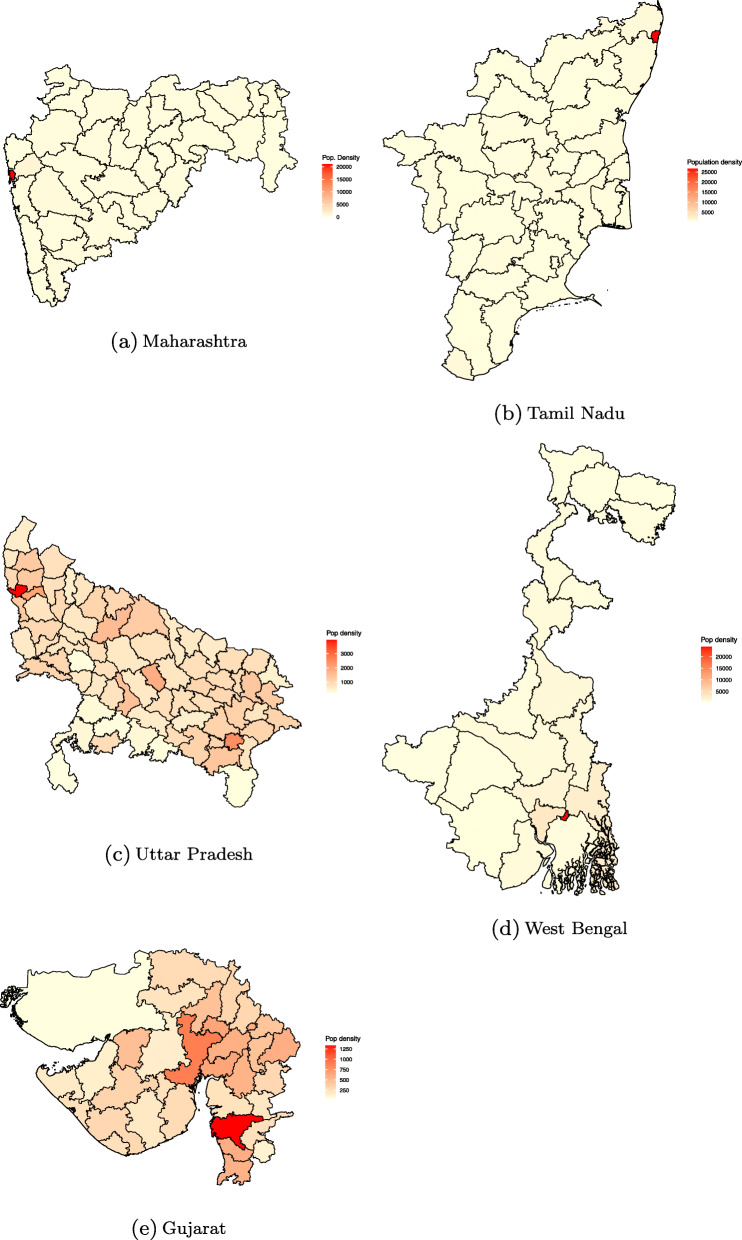
District-wise population density in selected states. These maps were created by the authors using R, packages *ggmap*, *maps*, *maptools* and *rgeos*

**Fig. 5:**
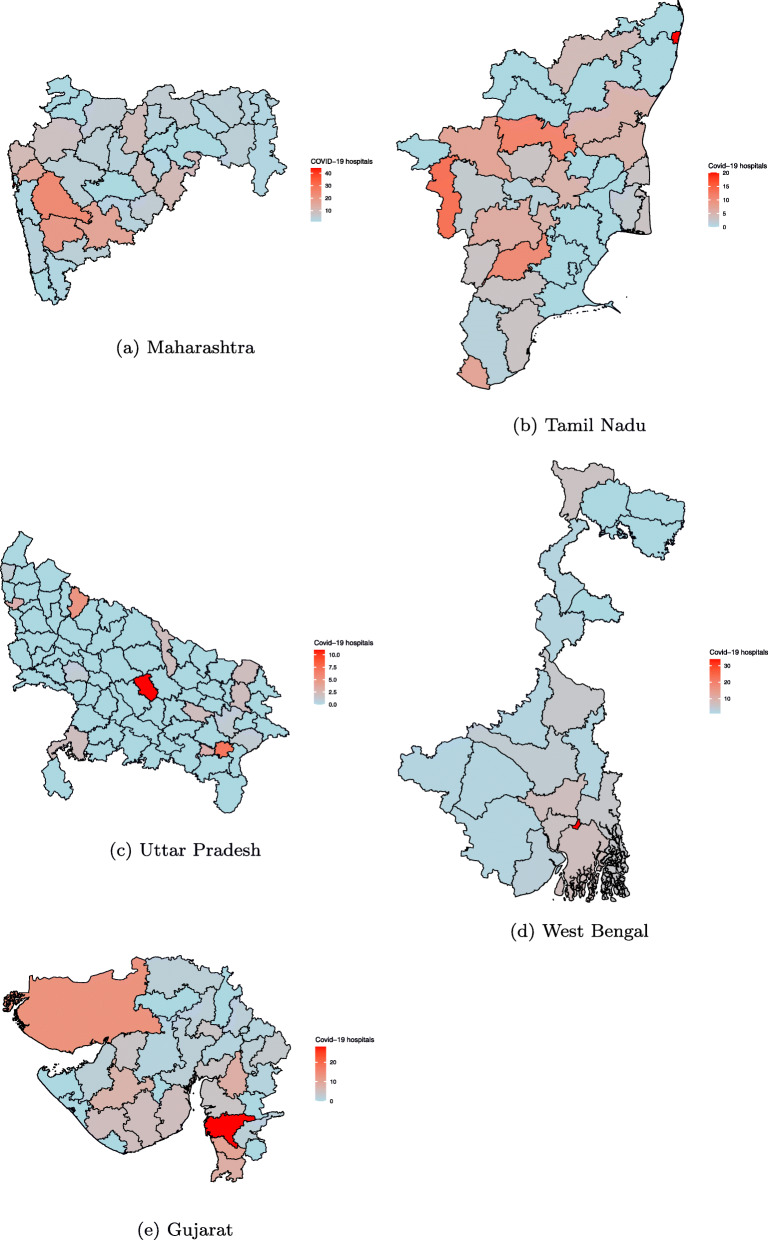
Distribution of COVID-19 hospitals in districts of selected states. These maps were created by the authors using R, packages *ggmap*, *maps*, *maptools* and *rgeos*

The population density map shows a similar pattern across districts in the states, refer Fig. 4. However, the distribution of number of COVID-19 hospitals in the districts vary from the distribution of confirmed COVID-19 cases, refer Fig. 5. The distribution of hospitals is much less skewed than the population density and COVID-19 cases. The state government and the central has been proactively trying to augment hospital facilities. The almost even distribution of the COVID-19 special hospitals in districts is a result of those initiatives.

**Fig. 6:**
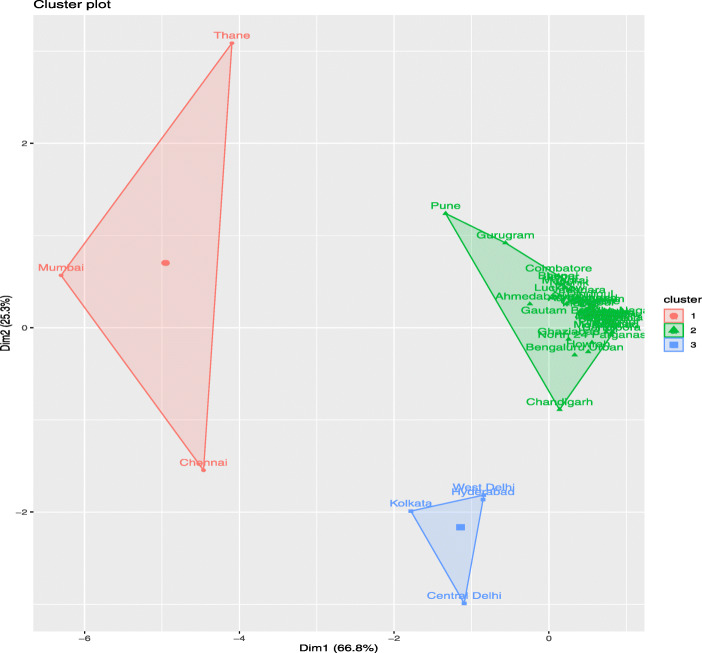
Visualization of three clusters generated from k-means clustering

The k-means clustering leads to three homogeneous clusters. The first of these, which include Mumbai, Thane and Chennai, have large number of confirmed COVID19 cases, number of hospitals as well as high population density. The second cluster has the lowest number of confirmed cases, COVID-19 hospitals and population density among the three. It includes cities like Kolkata, West Delhi, Central Delhi and Hyderabad. Unlike the first two clusters, cluster 3 has very high population density, but low number of COVID-19 hospitals and confirmed COVID-19 cases. Table 4 presents the centroids of each cluster, the central tendency values for each variable in each cluster. A diagrammatic representation is shown in Fig. 6.

**Table 4:** The table provides centroids of each cluster for the three variables *Confirmed*, COVID-19 *hospitals*, and *Density*

Variables	Cluster 1	Cluster 2	Cluster 3
Confirmed	64007	3151.5	3968.5
COVID hospitals	32	5.95	7.75
Density	23102.5	1166.9	22442.75

Thus it is observed that the burden on the healthcare system is maximum in the cluster one districts, while with their high density but low confirmed cases cluster three comprises of districts which have been effective in controlling the disease.

**Fig. 7:**
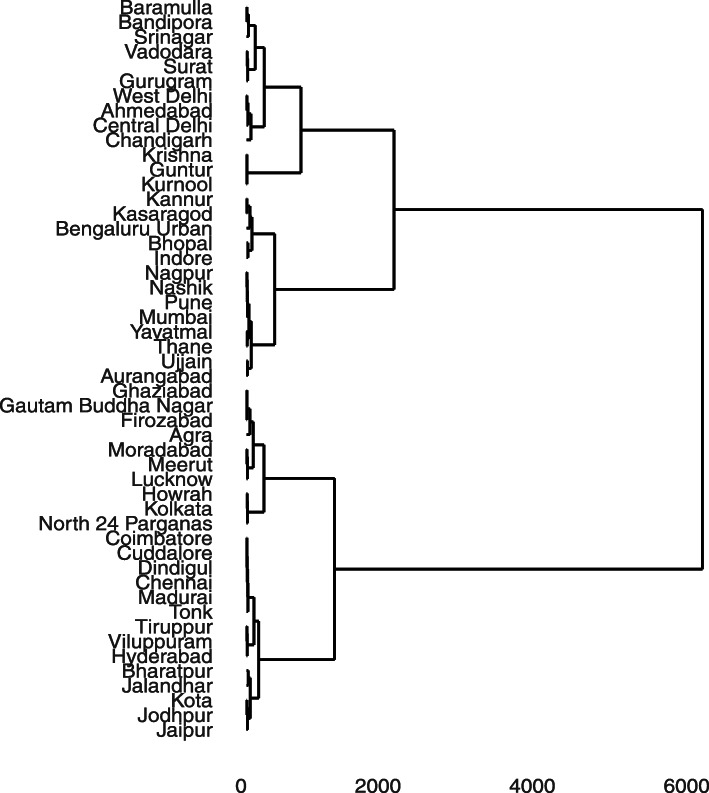
The dendrogram above is a result of hierarchical clustering. Based on the number of active positive cases of COVID-19, fifty worst hit districts of India were used to create clusters of homogeneous districts. Six clusters were created, demarcated in the diagram with the boxes around clusters of districts that had similar number of active positive COVID-19 cases

The hierarchical clustering (as shown in the dendrogram below in Fig. 7), done to show the closeness of two districts in terms of the above three parameters, corroborates our findings from the k-means clustering.

### Nizamuddin : a case study

In the wake of the global crisis due to COVID-19, almost all affected countries, have seen an exponential growth in the number of confirmed case count. In most of such countries, doctors discovered a gaggle of individuals who got infected at one place and mostly at around the same time. Such groups are termed *clusters*. In India one such case has been identified at the Nizamuddin basti in Delhi with its highly dense population. There have been numerous studies over the years, [17], that describes this area of Delhi as one of the more populous with almost 1500 households and a large floating population as well. Another study [18] discusses the development of the highly populated Nizamuddin basti around Hazrat Nizamuddin Auliya mosque. It is this locality that has been identified as a massive *cluster*, after a religious congregation held in mid-March led to COVID-19 spread among the attendees; at least 130 cases have been identified as having originated from this cluster.

As a special case study, we’ve used the SEIQHRF model to simulate the spread of the virus through infected individuals from the Nizamuddin *cluster*. A stochastic individual contact model (ICM) is employed to simulate the baseline projections of the timeline of establishment period of the virus, illness duration and survival time of the case fatalities. In the five panels of Fig. 8 we provide general statistical properties of the model in relation to some of the major parameters estimated.

1. The incubation period for the virus has a median of about 10 days and in few cases could reach 20 days or more.

**Fig. 8:**
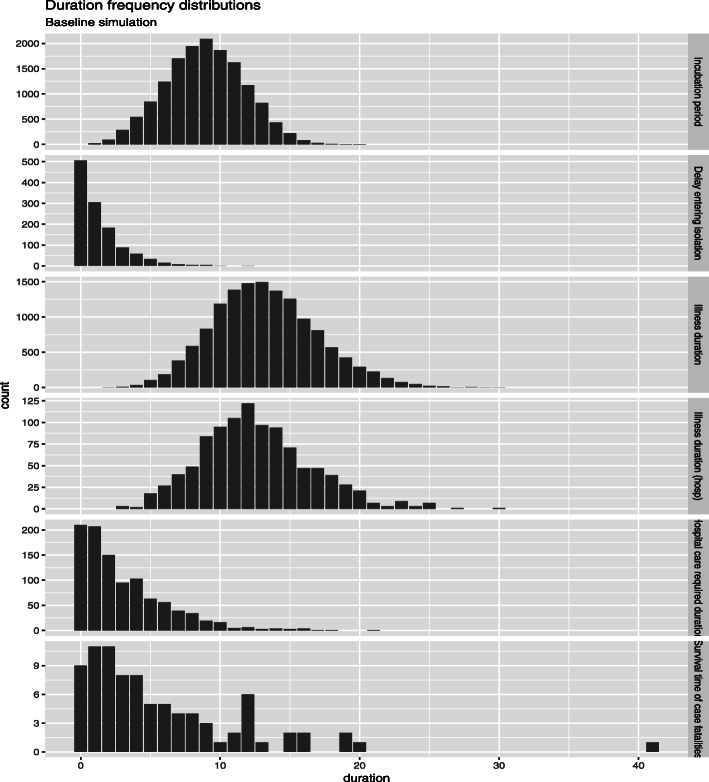
Nizamuddin tablighi cohort duration frequency distribution
2. For some, isolation started much later, could be as long as 10 days. However, many people took less than a couple of days before they started to isolate themselves because of some COVID-19 like symptoms.
3. Illness duration seems span around 25 days. Although this may vary based on the individuals comorbid conditions.
4. Hospital care duration is about 10 to 15 days mostly, which seems to match actual observations.
5. Survival time of case fatalities is seen to be mostly between two to five days. However few fatal cases have had a survival time as high as 20 days.

**Fig. 9:**
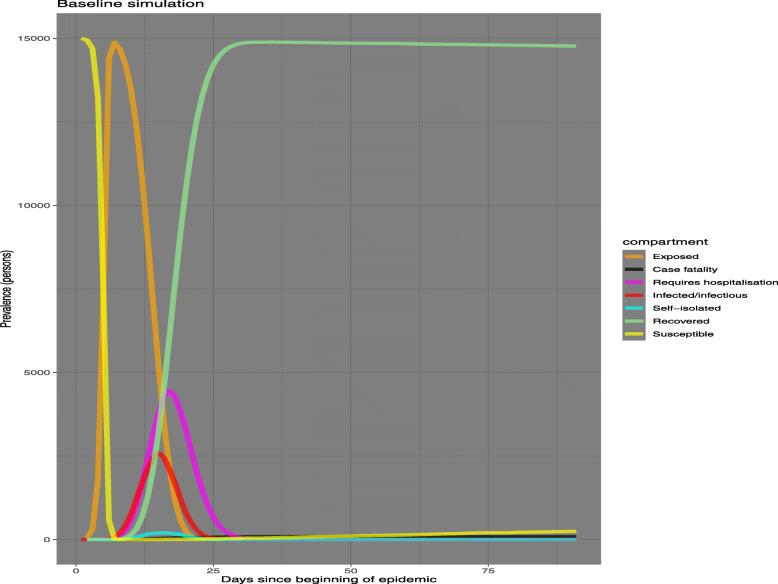
Prevalence simulation of COVID-19

The next plot shows the prevalence of COVID-19 among the Nizamuddin population in the various compartments of the SEIQHRF model. Prevalence is the number of people in each compartment at each point in time (each day). Given the dense nature of the population in the Nizamuddin basti the *act* parameter (average number of exposure events between infectious individuals and susceptible individuals) for this area is quite high making it a hotbed for the virus spread within the community. From Fig. 9 we can see how peaked the distribution of exposed individuals is in the neighbourhood. Also due to the delayed signs of this viral infection among individuals, the ones living in these densely populated areas unknowingly pass on the virus to others. The prevalence of cases where self-isolation by individuals is undertaken has a comparatively low peak, thus isolating oneself when symptoms are visible reduce the risk for others. The model estimate that from 15,000 susceptible people, almost as many as 15000 other people are potentially exposed during the 3 days event, and from this almost 15000, about 4000 are potentially infected and require hospitalization. However, due to the delayed signs of the infection causes possible delays in the people getting to the hospital for checking, which causes about 20 days delay in the infected person to be hospitalized. Furthermore, the number of people who went to self-isolation is predicted to be very small.

**Fig. 10:**
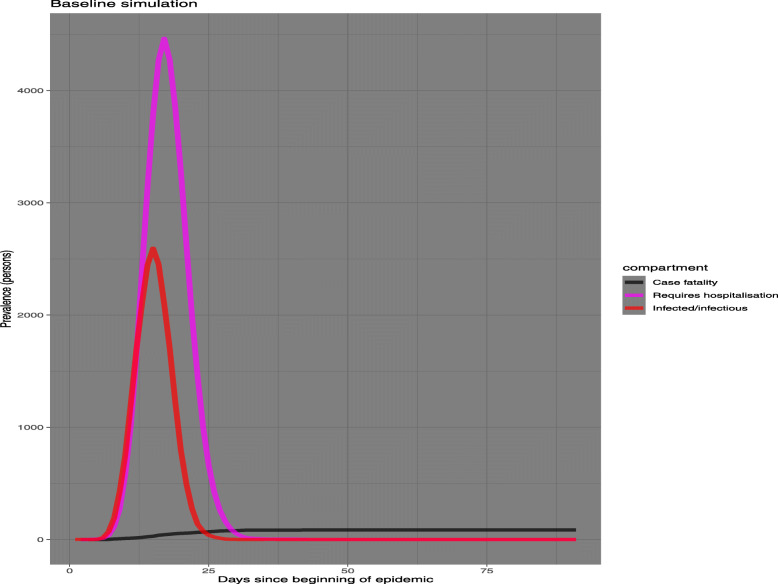
Prevalence simulation COVID-19 confirmed cases

In Fig. 10 we have isolated the infected, hospitalized and fatal compartments to look at them more closely. It shows that of the almost more than 4000 required hospitalisation, although with a delay of almost 15 days. The model used here predicts the prevalence in each compartment; shows the number of individuals in each compartment who have the condition during the time period under study. As we have stated before, sometimes the delayed signs of the infection cause a lag between the time infection is identified and it is serious enough to require hospitalisation. The time period of this study (second phase of lockdown in India) thus includes individuals for whom the infection was identified earlier than the present time but it aggravated enough to require hospitalisation much later. Thus making the predicted number larger than the size of the infected cohort. There were more than 2500 people infected. However, the number of fatal cases remained low, around 150.

Apart from the baseline scenario, we have tried to follow the path of prevalence in each compartment after the implementation of several interventions.

**Fig. 11:**
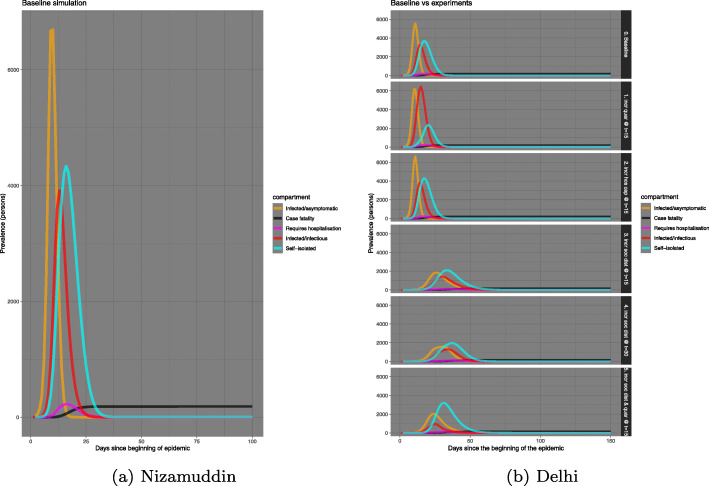
Comparison of prevalence between Nizamuddin cohort and Delhi
- *I*_2_: Starting Social Distancing from 15th April, 2020: The Government of India announced a second phase of lockdown from 15th April, 2020 till 9th May, 2020 as an intervention to stop the COVID-19 pandemic. Hence, as an image of Social Distancing, we gradually reduced the frequency of exposure events from 20 to 10.
- *I*_3_: Having introduced further social distancing from 15th April, 2020, we also introduced another intervention by increasing the hospital bed capacity to triple the initial number considered during the first 15 days of lockdown phase 2.
- *I*_4_: Imposing more severe social distancing by the end of second phase of lockdown, thereby further reducing the number of exposure events from 10 to 5.
- *I*_5_: Imposing a combination of increased social distancing and quarantining during the first 15 days of lockdown 2.

Some of these administrative interventions are implemented in the simulation of the Nizamuddin cluster and represented in Fig. 11a. A side-by-side comparison of similar implementation in Delhi is in Fig. 11b. A list of interventions considered in this study is;

- *I*_1_: Increase quarantining of symptomatic individuals during the first 15 days after the beginning of second phase of lockdown, i.e. 15th April 2020.

We will thereafter refer to the interventions as *I*_*i*_ for *i*=1,2,3,4,5.

The same set of interventions have been used for other states as well, where attendees from Nizamuddin have returned after the convention.

The baseline plot shows that the number of infected/infectious individuals in the Nizamuddin cluster is close to 10,000, almost double of that in Delhi. This can mostly be s result of the very fact that in an exceedingly dense geographic area there is a larger propensity for people to mingle with more number of individuals, thus increasing the prevalnce of the spread of COVID-19 manifold.

In both Nizamuddin cluster and Delhi, the curve of infected/infectious individuals have flattened with implementation of the above interventions. Among the administrative mediation, increasing social distancing as well as self-isolation starting on day 15, after the beginning of the disease outburst, produces the best results in flattening the disease spread curve. However, a comparison shows that the Nizamuddin cluster increased the number of confirmed cases and also the number requiring hospitalisation. The numbers have almost doubled from that of Delhi.

The Nizamuddin cluster was discovered in mid-March and at least 130 cases in India have been identified as having originated from this cluster. There was then a second wave of infectious individuals identified in several other states that were linked to the Nizamuddin cluster. Tamil Nadu, Delhi, Telengana, Gujarat, Maharashtra are a some of such states.

**Fig. 12:**
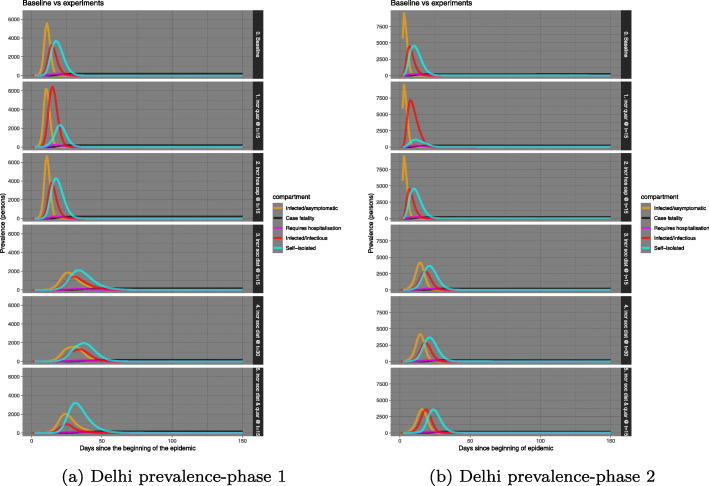
Comparison of baseline prevalence with those with interventions for Delhi

**Fig. 13:**
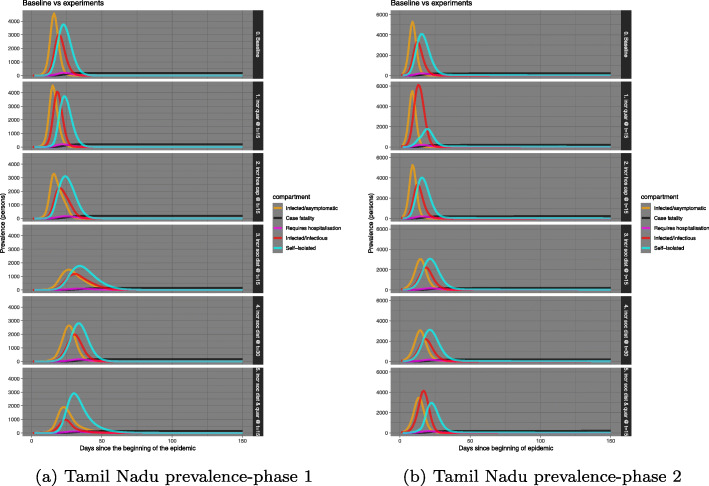
Comparison of baseline prevalence with those with interventions for Tamil Nadu

**Fig. 14:**
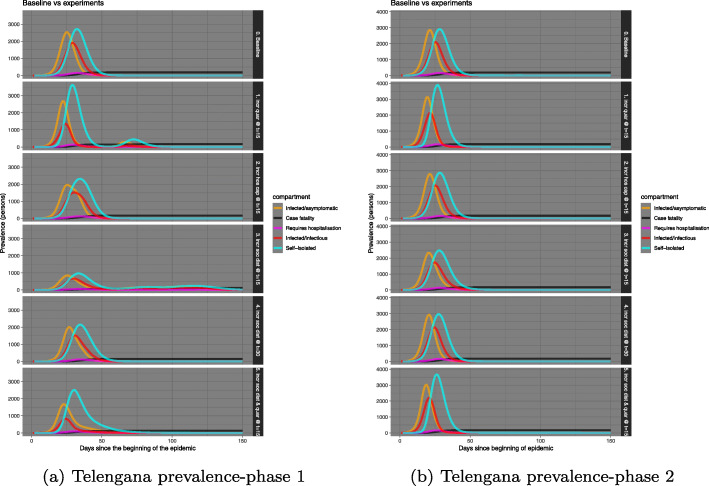
Comparison of baseline prevalence with those with interventions for Telangana

In each of the states above (Tamil Nadu, Delhi, Telengana) the number of infected individuals linked to Nizamuddin cluster are 72, 24 and 6 respectively. The comparison in sets of Figs. 12, 13, and 14 show that in the second phase the number of infected individuals had seen a multitudinous increase. Thereby, increasing the danger of disease spread within the respective states. However, the simulations reveal that the administrative interventions, if implemented strictly, flatten the curve of disease spread. Specifically, the best results are seen if social distancing is strictly practised by day 15 since the disease outbreak.

In Table 5 first panel, the maximum predicted numbers in each compartment is presented for Nizamuddin basti right after the Tablighi Jamaat congregation (from SEIQHRF model fitting, also shown in Fig. 11a). With the choice of parameters defined in “Choice of parameters” section, the baseline model is fitted, but *I*_1_ through *I*_5_ are the various interventions applied and the corresponding parameters have been modified accordingly. For instance, in the baseline case the projected number of infected cases was maximum around 5000, with the implementation of *I*_1_ i.e. increased advocacy of quarantining the maximum projected individuals infected increased even more (around 7500). However, for *I*_2_ through *I*_5_ this number decreased. Thus, social distancing, augmentation of hospital capacity and severe quarantining implemented together prove to be useful in flattening the curve. The second and the third panel are predictions from Delhi during the first phase of lockdown, starting point is 24th March 2020. The third panel shows the predicted number of individuals in each compartment after the attendees of Nizamuddin meeting dispersed. A comparison of infected individuals increased manifold between the first lockdown and after Nizamuddin congregation. The number of infected before Tablighi Jammat (1000) was the lowest for intervention *I*_5_ i.e. increased social distancing and quarantining. However, for the Nizamuddin cohort a severe imposition of social distancing worked better than any other intervention.

**Table 5:** List of variables used and the maximum predicted counts of each compartment for a cohort of Nizamuddin and Delhi residents

No.	Interventions	Infected	Susceptible	Requiring hospitalisation	Fatal
Nizamuddin basti:					
1	Baseline	5000	10000	200	50
2	*I*_1_	7500	10000	200	50
3	*I*_2_	4500	10000	200	50
4	*I*_3_	3750	6250	100	50
5	*I*_4_	3750	6250	75	50
6	*I*_5_	3800	5000	50	50
Delhi:phase 1 (before Tablighi Jamaat)					
1	Baseline	3100	5800	200	175
2	*I*_1_	6100	6100	225	180
3	*I*_2_	4000	6500	220	150
4	*I*_3_	1700	2000	125	150
5	*I*_4_	1200	1800	100	140
6	*I*_5_	1000	2000	125	150
Delhi:phase 2 (after Tablighi Jamaat)					
1	Baseline	5000	10000	240	200
2	*I*_1_	7300	6100	250	200
3	*I*_2_	4800	6500	240	200
4	*I*_3_	2700	2000	200	150
5	*I*_4_	2600	1800	200	150
6	*I*_5_	3750	2000	150	150

Similar to Table 5 we have also tabulated the maximum predicted number of individuals in each of compartment for Tamil Nadu and Telengana for both before and after phases of the Nizamuddin meeting, results shown in Tables 6 and 7. In case of Tamil Nadu (Table 6), a combination of social distancing and quarantining during the first lockdown phase visibly lowers the number infected (1000), however, after the attendees from Nizamuddin cohort returned to the various states, Tamil Nadu and Telengana were one of the largest clusters of COVID-19 infections. In the second phase therefore increasing the hospital capacity helped in flattening the curve of infected persons. With the augmented hospital capacity proves to be an effective intervention. For Telengana (Table 7), in both phases 1 and 2, i.e. before and after the Nizamuddin cohort returned to the state, augmented hospital capacity seems like the best among the five interventions. Commensurate with the number of susceptible people in the states we also look at the number of fatalities and the ones requiring hospitalisation. But, the records of the number of days of hospitalization are not publicly available by each patient, thus making if unreliable to infer much from the simulation on how long the period would be. We also cannot claim the fatality rates with too much certainty because there are many unknown parameters involved and not much data is available for the co-morbidity conditions of the patients either.

**Table 6:** List of variables used and the maximum predicted counts of each compartment for a cohort of Tamil Nadu residents

No.	Interventions	Infected	Susceptible	Requiring hospitalisation	Fatal
Tamil Nadu:phase 1 (before Tablighi Jamaat)					
1	Baseline	3000	4500	200	175
2	*I*_1_	4000	4600	200	200
3	*I*_2_	2200	3500	175	190
4	*I*_3_	1200	1500	100	150
5	*I*_4_	2000	2600	160	175
6	*I*_5_	1000	2000	120	150
Tamil Nadu:phase 2 (after Tablighi Jamaat)					
1	Baseline	3200	5200	220	200
2	*I*_1_	6000	5300	250	200
3	*I*_2_	3200	5300	220	250
4	*I*_3_	2200	3100	175	200
5	*I*_4_	3000	3000	175	200
6	*I*_5_	3500	3400	180	180

**Table 7:** List of variables used and the maximum predicted counts of each compartment for a cohort of Telengana residents

No.	Interventions	Infected	Susceptible	Requiring hospitalisation	Fatal
Telengana:phase 1 (before Tablighi Jamaat)					
1	Baseline	2000	2500	200	175
2	*I*_1_	1500	2200	225	180
3	*I*_2_	1500	2000	220	150
4	*I*_3_	600	1000	125	150
5	*I*_4_	1500	2000	100	140
6	*I*_5_	800	1600	125	150
Telengana:phase 2 (after Tablighi Jamaat)					
1	Baseline	2000	3000	160	180
2	*I*_1_	2100	3000	170	175
3	*I*_2_	2000	2800	155	175
4	*I*_3_	1800	2500	148	165
5	*I*_4_	2100	3000	152	175
6	*I*_5_	2200	3000	151	170

### Dharavi: a case study

Dharavi is considered Asia’s largest slum, one of the most densely populated areas in the world (with 2.6 lakh people per sq. km) and, now, also marked as a containment zone for COVID-19. When driving through the lanes and by-lanes of Dharavi, thronging with people, it is evident that the one necessary norm to prevent the spread of COVID-19, social distancing, is practically impossible to implement here. Most of the houses in the area are merely 10X10 feet, it is a challenge to keep people confined to such small area. In addition, there is a major problem with the common toilets that inhabitants have to use, which makes the containment of the virus an impossible task. Although the first case of COVID-19 in India was reported on the 30th of January, by middle of March only mere eight positive cases of COVID-19 were identified in Mumbai. All of these cases had a travel history abroad. However, by the end of March, the number of positive cases started growing exponentially. In Dharavi the first case was observed on the first of April, [19]. The index patient was a 56 year-old garment shop owner complained of high fever and cough. When his symptoms worsened he was admitted to the hospital where he succumbed to the disease before civic officials could talk to him about the people he might have come in contact with. So the officials began their contact-tracing exercise. It came to light during their investigation that a few days before the garment shop owner started showing the signs of COVID-19 infection, he had hosted a party with some people in his house, and all these guests were attendees of the religious event that took place in mid-March in Nizamuddin area of Delhi.

**Fig. 15:**
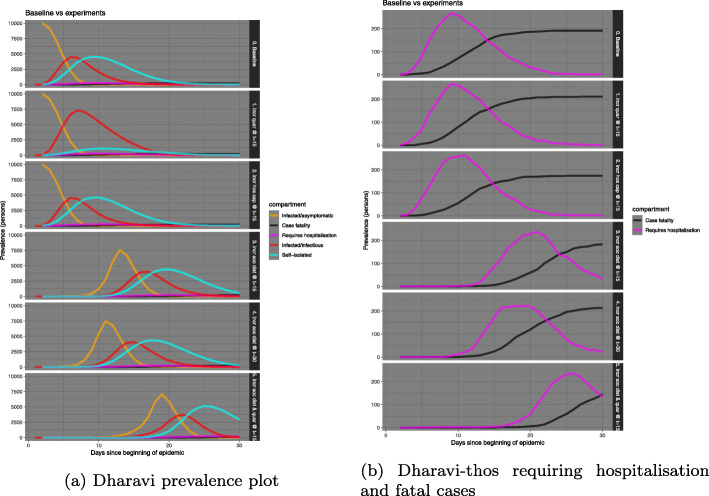
Prevalence plots in various compartments for Dharavi

Other than the constraints of space that make the inhabitants more exposed to the virus, most COVID-19 positive cases in Dharavi have been found to be asymptomatic. The silent carriers living in the area were unaware of how many people they were infecting with the virus. A simulation run using the SEIQHRF model produces the number of infected/ asymptomatic, infectious, self-isolated, hospitalised and fatal cases in Dharavi (Fig. 15).

Due to the very high *act* parameter value for Dharavi the SEIQHRF model algorithm is used to simulate the scenario for a period of 30 days. The results illustrate the predicted course of COVID-19 and the impacts of various experimental interventions. Dharavi has especially been a challenge for the Maharashtra government, slowly turning out to be the COVID-19 capital of the state. From Fig. 15 it is seen that the prevalence rate among the residents was very high since the beginning of the outbreak. The lack of space to practice social distancing and the use of common toilets, exposed them more to the risk of acquiring the virus. In the baseline model the number of infectious/asuymptomatic patients is as high as almost 10,000. However, measures like increased social distancing and self-quarantine is expected to help in flattening the curve to some extent. From the panel on the left we can see that with increase in social distancing and quarantining the number of infected/asymptomatic has come down by almost 2500. There is also a delay in the prevalence number reaching it’s peak, which is almost 10 to 15 days. Also the panel on the right reveals predicted number of people requiring hospitalization. Dharavi alone predicts a requirement of 200 hospital beds for hospitalization. As, of 11th July 2020 the total number of positive cases are 2359. The fatality rate in Dharavi increased from about 3% to 4.1% over a period of two weeks between May 5th and May 20th.

Amidst all the challenges, Dharavi has emerged as one of the few successes in containing the spread of COVID-19 with 1952 recovered cases of its 2359 confirmed and only a handful 166 remaining active. According to the Brihanmumbai Municipal Corporation officials, the steps taken in Dharavi can be defined by four T’s- tracing, tracking, testing and treating. Almost as many as 47500 houses were screened, while 14970 people were screened in mobile vans. In the study done by [20], the author states that the WHO has also acknowledged Dharavi’s success in controlling the spread of COVID-19 and mentioned that it should be seen as an example across the world. The WHO has also commended the Dharavi model which is mostly based on community engagement, testing, tracing, isolating, and treating in order to break the chain of transmission. Quarantine facilities were ramped up with the use of schools, marriage halls and sports complexes. The strict implementation of lockdown and an endeavour to treat all COVID-19 patients in Dharavi itself proved successful in containing the infection. A proof that these interventions were fruitful is the fact that while in April, the doubling rate in Dharavi was 18 days, it gradually improved to 43 days in May and slowed down to 108 and 430 days in June and July respectively. In a recent press meet officials from WHO cited Dharavi as one of the few cases around the world where a massive breakout of the infection could still be brought under control, [21]. In [20] the author reports that he municipal administration of Mumbai very aptly called the Dharavi model as “chasing the virus” rather than waiting for people to report it. According to [22] under the able leadership of the Mumbai municipality, Brihanmumbai Municipal Corporation (BMC), private medical practitioners, social activists, community leaders, and non-governmental organisations were roped in to battle the pandemic on a war footing. Measures were taken to give older people special attention. Most symptomatic cases were treated at community centres, except when critical and time was invested in trust building efforts to gather public support for containment of the disease. In this study the authors report that unlike the traditional approach of passive screening as undertaken elsewhere in India, the civic authorities of Brihanmumbai Municipal corporation adopted a proactive screening strategy in Dharavi and went door-to-door looking for COVID-19 suspects. Which supports the claims made by the municipal administration of Mumbai, that they were chasing the virus [20].

## Discussion

The various scenarios analysed in this paper introduce the various interventions undertaken and compare the course of COVID-19 under each of them with a baseline model. To summarise the outcomes some important observations made are as follows: In the baseline model, the results are from our model fitting on a hypothetical population of 1000 people. The findings are summarized as follows;

- Time taken for the COVID-19 pandemic to abate is about two months.
- As the population density of Delhi is very high, many people are infected, although asymptomatic.
- We see typical exponential behaviour, although these are prevalence numbers, not incidence. The prevalence counts tends to start with an exponential growth; however they then diminish.
- The number of people who require hospital facilities is not too large.
- Although the growth rate is much lower, the number of cases in the fatality compartment is increasing consistently.

But the various interventions have varied effect on the spread of COVID-19. In most of the states in India the best results are seen in implementation of early social distancing and quarantine of the infectious individuals. In states where a lot of attendees from Nizamuddin had returned, extra care was taken to isolate them and that to some extent alleviated the risk of infection spread. We see an exponential behaviour in the growth of prevalence in the panels, although these are not incidence. Prevalence starts with an exponential growth but then diminishes. That’s what we are seeing here, so that’s a good sign.

In case of Nizamuddin, the highest frequency is seen between day 5 and 7, the individuals for whom the disease turned fatal mostly survived for five to seven days. The attendees of the gathering in Nizamuddin then exposed some of the states/UTs, like, Delhi, Tamil Nadu and Telangana to a second wave of COVID-19 infection. A side-by-side comparison of prevalence in these states during the first and second wave show a significant growth in number of COVID-19 positive cases.

Dharavi is one of the unlikely success stories in India. In spite of being one of the largest slum areas of Asia, the Brihanmumbai Municipal Corporation has managed to control COVID-19 spread in an area merely 2.1 *km*^2^ housing almost a population of 10,00,000. The four T’s- tracing, tracking, testing and treating have worked wonders for the slum and when large metropolises around the world like New York, Los Angeles, London reeling under the COVID-19 attack, a slum in Mumbai India has managed to reduce the reproduction number to a record low. In comparison to Dharavi, Mumbai a city which Dharavi is a part of, has suffered more severe fate at the hands of the deadly virus with almost 2.61 lakh active cases as of 6th November 2020.

## Limitations and future studies

The main limitation of this study is that the COVID-19 scenario is still very dynamic and evolving with each passing day. Predicting the true path that the virus is going to take, is difficult if the parameters in the model are incorrectly specified. And with the ever changing COVID-19 landscape, it is one of the major challenges in model fitting. With new strains of the virus emerging, the unavailability of disaggregate level data of the confirmed cases makes modelling a challenge. There is also lack of available data on co-morbid conditions of the patients hospitalised with COVID-19 along with lack of data on actual hospitalisation. This adds to the already existing impediments in the way of modelling the path of the virus spread. Availability of such data would enable a more robust forecast of the number of confirmed COVID-19 cases. The data would also help in early detection of COVID-19 clusters which would be helpful for executing public health policies. Also the government has been trying to implement new policies to curb the spread. During the time-line of this study we have only looked at the first three months of the pandemic outbreak in India. As an extension of this study we would like to tap into the increasing data on COVID-19 and follow the path of the virus in the more recent unlock phases in India. Also a longitudinal analysis of COVID-19 data from the worst hit districts and following them over the months can give us more useful insights.

## Conclusion

The top 50 districts of India where most severe outbreak of COVID-19 was seen are analysed through cluster analysis using their respective population densities and number of COVID-19 hospitals. The results helped identify the cities where similar pattern of disease outbreak was seen as well as the ones with homogeneity in terms of COVID-19 hospitals and population density. An in depth look at the various mediations undertaken to curb the growth of COVID-19 in India have shown different levels of improvement in flattening the disease curve. As the attendees of Nizamuddin congregation returned to their home states, like Tamil Nadu, Telangana, and other parts of Delhi the second wave of surge in confirmed case numbers is seen. A comparison between the first and second wave of COVID-19 infection in select states also support this finding. A further study of Dharavi, one of the success stories of the biggest slums in Asia, is undertaken and the strategy of 4 T’s-tracing, tracking, testing and treating has shown to reduce the spread of the disease dramatically.

## Data Availability

The datasets used and/or analysed during the current study are simulated based on the epidemiology model used in this study. The data used and prevalence plots generated thereafter have been simulated and created using the R programming language, packages *EpiModel*, and *incidence*.

## Abbreviations

COVID-19: Corona Virus Disease 2019
SIR model: Susceptible-Infectious-Recovered model
SEIR model: Susceptible-Exposed-Infectious-Recovered model
SEIQHRF model: Susceptible-Exposed-Infectious-Quarantine-Hospitalised-Recovered-Fatal model
